# A critical use of peripherally inserted central catheters in the intensive care unit

**DOI:** 10.62675/2965-2774.20260237

**Published:** 2026-03-03

**Authors:** Viviane Cordeiro Veiga, Pedro Henrique Rigotti Soares, André Kalil, Pedro Póvoa

**Affiliations:** 1 BP - A Beneficência Portuguesa de São Paulo São Paulo SP Brazil BP - A Beneficência Portuguesa de São Paulo - São Paulo (SP), Brazil.; 2 Hospital Nossa Senhora da Conceição Porto Alegre RS Brazil Hospital Nossa Senhora da Conceição - Porto Alegre (RS), Brazil.; 3 University of Nebraska Medical Center Nebraska United States University of Nebraska Medical Center - Nebraska, United States.; 4 Hospital S Francisco Xavier Centro Hospitalar de Lisboa Ocidental Lisboa Portugal Hospital S Francisco Xavier, Centro Hospitalar de Lisboa Ocidental - Lisboa, Portugal.

## INTRODUCTION

Obtaining central venous access is a priority in the management of every critically ill patient, since it provides a more stable access than peripheral vascular access and allows the administration of several drugs simultaneously (multi-lumen catheters), even irritating medications, and the performance of techniques (such as dialysis with a proper catheter). Besides, they can also be used as part of hemodynamic monitoring.^(1)^

Currently, a central venous access can be obtained with a peripherally inserted central catheter (PICC) or a central venous catheter (CVC). The PICC is placed using a modified Seldinger technique, usually in the basilic vein. They have 1 or 2 lumens, and they should be cut according to the distance from the insertion site to the right atrium (on average, 52cm on the right and 56cm on the left). The CVC is placed using the Seldinger technique in a central vein (internal jugular, subclavian, or femoral), it has 1 to 5 lumens, and the length is usually between 15 - 25cm.^(1-3)^

The objective of this article is to critically analyse the use of PICC in critically ill patients, discussing current evidence, potential benefits, limitations, and implications for clinical practice.

### Costs

In Brazil, a double-lumen CVC costs approximately R$ 227,00, while a PICC line can cost around R$ 950,00, making it over four times more expensive. Internationally, this discrepancy can be even more pronounced, reaching up to a tenfold increase in countries like Portugal.

### Complications

#### Mechanical complications

Venous accesses are associated with complications, some related to insertion (e.g., mechanical complications, such as pneumothorax), and others to their maintenance (e.g., infection, vein thrombosis, and lumen occlusion).

Peripherally inserted central catheters are associated with a lower risk of mechanical complications compared with centrally inserted CVCs. Notably, they carry no risk of pneumothorax and are considered safer, particularly when performed under ultrasound guidance.^(4,5)^ Nonetheless, complications such as catheter tip malposition and occlusion remain prevalent. Incidence rates range from 6.9% to 31% for occlusion and 9.3% for tip dislodgement. Overall complication rates vary from 11.2 to 30 per 1.000 catheter-days, with an average PICC dwell time of 27 days.^(1-3)^

#### Thrombotic complications

The emergence of PICC thrombosis, a dangerous complication, is a significant concern due to its incidence. The incidence of PICC thrombosis is around 1% to 9% of the total number of catheters inserted.^(1,2)^ This highlights the most dangerous complication in PICC in critically ill patients and emphasizes the need for vigilance.^(1-3)^

Meta-analysis published by Chopra et al.,^(6)^ including more than 29,000 adult patients, demonstrated a high risk of venous thrombosis compared to central venous catheters (OR 2.55; 95%CI 1.54 - 4.23; p < 0.001), with a weighted frequency of PICC-related deep vein thrombosis of 6.7% (95%CI 4.7 - 8.6).

A Cochrane review including 12 trials and 2,913 patients evaluated different PICC materials and designs to reduce catheter failure and complications. However, the available evidence was insufficient to determine whether any specific material or design is superior in preventing such complications.^(7)^

#### Infectious complications

Infectious complications are expected with all intravenous catheter lines due to the need for skin barrier disruption.^(8)^ However, the magnitude of the risk may vary depending on the type of intravenous catheter line. A systematic review of 23 studies with 57,250 patients found that PICC was associated with central line-associated bloodstream infection (CLABSI) reduction in the outpatient setting, but not in the inpatient setting.^(6)^ When comparing 1,730 PICC days with 637 CVC days inserted in the intensive care unit, only one central-line-associated bloodstream infection occurred in a PICC line.^(9)^ Although PICCs are associated with a significant reduction in the incidence of infectious complications (OR 0.52; 95%CI 0.40 - 0.66; I2 = 0%), this finding warrants critical analysis. In clinical settings with already low CLABSIs rates, the economic benefit of adopting PICCs may be attenuated, as the reduced infection risk might not offset the device's higher upfront cost. Therefore, cost-effectiveness should be evaluated in the context of each institution's baseline infection rates and resource availability, which is consistent with another study showing lower CLABSI rates with PICC lines compared to CVCs (OR 0.38; 95%CI 0.16 - 0.91).^(10)^

Table 1 summarizes the comparative risks of thrombotic and infectious complications between PICCs and CVCs, highlighting the main odds ratios reported in recent studies.

**Table 1:** Comparative risk of complications between peripherally inserted central catheters and central venous catheters in critically ill patients

Complication	PICC versus CVC	Odds ratio (95%CI)
Infectious complications (CLABSI)^(6)^	Lower with PICC	0.52 (0.36 - 0.76)
Symptomatic thrombosis^(6)^	Higher with PICC	2.55 (1.54 - 4.23)
Mechanical complications^(8)^	Lower with PICC	Not quantifiable, but negligible risk of pneumothorax
Catheter failure^(7)^	No significant difference	OR ~1.0 (varied across studies)

1 PICC - peripherally inserted central catheter; CVC - central venous catheter; CLABSI - central line-associated bloodstream infection; OR - odds ratio.

## CONCLUSIONS

Overall, the published evidence remains limited regarding the risks of peripherally inserted central catheter use compared to central venous catheter in the intensive care unit, but suggests that the risk of thrombosis is variable and the risk of bloodstream infection is minimal. Additionally, differences in definitions of catheter-related bloodstream infection and thrombosis, along with varying sample sizes and a small peripherally inserted central catheter population in the intensive care unit, indicate that more studies are still necessary to define the precise risks of infection and thrombosis with of peripherally inserted central catheter lines in the intensive care unit.

## Data Availability

The contents underlying the research text are included in the manuscript.
